# Viral metagenomics of fecal samples from non-human primates revealed human astrovirus in a chimpanzee, China

**DOI:** 10.1186/s13099-016-0140-2

**Published:** 2016-11-08

**Authors:** Xiaochun Wang, Jinxin Wang, Chenglin Zhou, Shixing Yang, Quan Shen, Wen Zhang, Dunwu Qi

**Affiliations:** 1 Department of Pathogenic Biology, School of Medicine, Jiangsu University, Zhenjiang, 212013 Jiangsu China; 2 Sichuan Key Laboratory of Conservation Biology for Endangered Wildlife, Chengdu Research Base of Giant Panda Breeding, Chengdu, 610000 Sichuan China; 3 Department of Laboratory Medicine, Jiangsu Taizhou People’s Hospital, Taizhou, 225300 Jiangsu China

**Keywords:** Astrovirus, NHP, Phylogenetic analysis, Viral metagenomics method

## Abstract

**Background:**

Human astroviruses (HAstVs) are commonly identified worldwide as important aetiological agents of acute gastroenteritis in all age groups. More and more evidences challenged the paradigm that AstV infections are species-specific. Yet to date, AstVs associated with human infections have not been detected in any animal hosts.

**Results:**

Viral metagenomics methods were used to detect viral nucleic acids in fecal samples from 69 captive non-human primates (NHPs) from three zoos in China. Sequence reads showing high similarity to astrovirus MLB2 were found in feces from a chimpanzee with diarrhea. The complete genome of this astrovirus was determined and deposited in the GenBank under accession number KX273058 (named SAstV-nj). Phylogenetic analysis based on complete genomes revealed that SAstV-nj was closely related to and shared >98% nucleotide sequence identity with the previous human astrovirus MLB2 strains.

**Conclusions:**

This study suggested that MLB2-related astroviruses might have the potential of cross-species transmission between human and NHP.

## Findings

Astroviruses (AstVs), belonging to the family *Astroviridae,* are non-enveloped, positive-sense and single-stranded RNA viruses with icosahedral particles approximately 28–30 nm in diameter.

AstVs cause gastroenteritis in the young children, but in animals their association with enteric diseases is not well documented, with the exception of turkey and mink AstV infections [1]. The number of AstV-infected animal hosts has rapidly expanded in recent years. AstVs can infect at least 30 species of mammals including humans, sheep, cows, pigs, dogs, cats, red deer, mice, minks, bats, cheetahs, brown rats, roe deer, sea lions, dolphins, rabbits and NHPs [2, 3]. AstV infections were previously thought to be species-specific [4, 5], but these recently identified human AstVs, including HAstV-MLB1-3, HMO AstVs A, B, and C, and HAstV-VA 1-4, were genetically much closer to AstVs from animals than they are to the canonical HAstVs and HAstV1-8 [3]. Similar observations were reported for AstVs detected in pigs, bats, California sea lions, sheep, minks and turkeys [3]. Detection of potential human-animal AstV genetic recombination suggested that the species barrier may have been crossed at some point [6, 7]. More and more evidences challenged the paradigm that AstV infections are species-specific. Yet to date, AstVs associated with human infections have not been detected in any animal host.

In this study, using viral metagenomics, an AstV strain which shows close relationship with the AstV MLB2 strains from human based on the complete genome sequence was detected in the fecal sample of a chimpanzee having diarrhea.

From Sept. 2010 to May 2013, 69 fecal specimens were collected from 69 NHPs from three zoos in Eastern China. Viral metagenomics method as previously described [8] was used to detect viral nucleic acids in these fecal samples. Briefly, fecal samples were suspended in DPBS, vortexed for 10 min, and then centrifuged at 12,000 rpm for 10 min. The stool suspensions were collected in new 1.5 ml centrifuge tubes. Seven pools were randomly generated each of which contained ten fecal samples except the last one which contained nine samples. After low speed centrifugation and filtration, the pooled sample suspensions were treated with DNase and RNase, to reduce levels of NHP nucleic acids while viral genomes are protected with the viral capsid from digestion. Seven libraries were then constructed using Nextera XT DNA Sample Preparation Kit (Illumina) and sequenced using the Miseq Illumina platform with 250 bases paired ends, with a distinct molecular tag for each pool. Paired-end reads were decoded using vendor software from Illumina. An in-house analysis pipeline running on a 32-nodes Linux cluster was used to process the data. Clonal reads were removed and low sequencing quality tails were trimmed using Phred quality score ten as the threshold. Adaptors were trimmed using the default NCBI BLASTn parameters of VecScreen [9] with specialized parameters designed for adaptor removal. The cleaned reads were de novo assembled by SOAPdenovo2 version r240 using Kmer size 63 with default settings [10]. The assembled contigs, along with singlets were aligned to an in-house viral proteome database using BLASTx. The significant hits to virus were then aligned to an in-house non-virus-non-redundant (NVNR) universal proteome database using BLASTx. Hits with more significantly adjusted E-values to NVNR than to virus were removed.

**Fig. 1:**
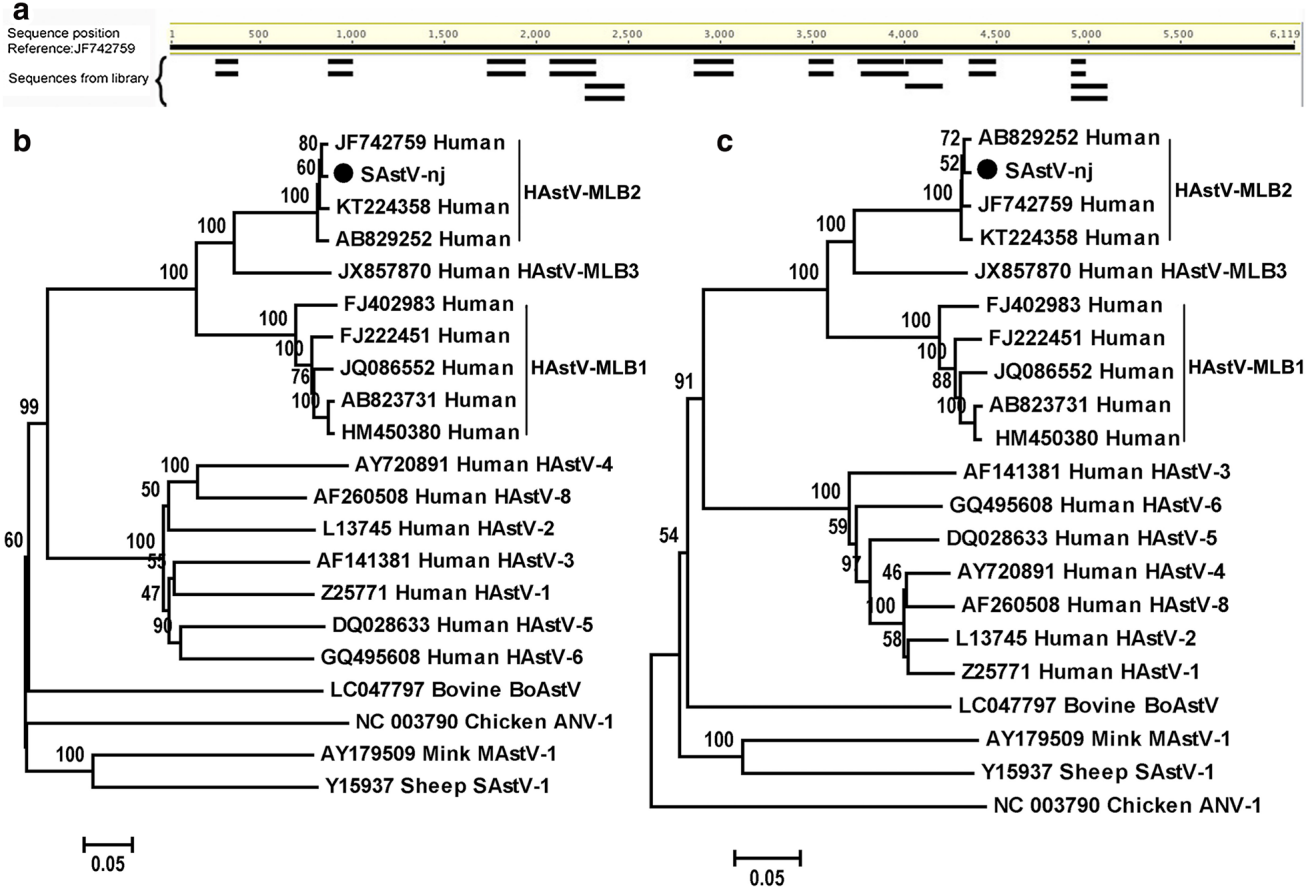
Mapping astroviral reads in the raw library data over the referenced genome of JF742759 (**a**) and neighbor-joining phylogenetic trees using MEGA6 (**b**, **c**). **b** was the phylogenetic tree based on capsid protein gene sequences. **c** indicated the phylogenetic tree based on RNA-dependent RNA polymerase gene sequences. *Black dot* indicated the SAstV-nj identified in this study. GenBank accession numbers were shown

The Illumina MiSeq 2 × 250 base runs of the seven libraries generated raw sequence reads of 888598, 718368, 687546, 545952, 434548, 896488, and 476396, respectively. When the viral sequence reads from the library that contained 888598 raw sequence reads were analyzed, 24 sequence reads showed >97% nucleotide sequence identity to those of human AstV MLB2 strains available in GenBank. In order to investigate the coverage of these AstV sequence reads in the AstV MLB2-positive library, the raw sequence data of this library was then aligned to a representative AstV strain MLB2 (GenBank no. JF742759) which was used as a reference genome by “map to reference” in Geneious software (version 8.1.8). Results indicated that the 24 astroviral reads were mapped to 9 different regions of JF742759, covering 31% (1869/6119) of its complete genome (Fig. 1a). PCR primers were then designed based on the largest contig, and PCR screening was performed to detect this virus in the 69 fecal samples. Results indicated that only one positive sample in the original AstV-positive library, which was from a chimpanzee with diarrhea. Viral metagenomic analysis revealed that the library positive for the AstV also contained viral reads showing similarity to simian adenovirus and Po-Circo-like virus. PCR using primers designed based on the contigs of the two types of viruses indicated that the AstV-positive sample was negative for the adenovirus and Po-Circo-like virus.

In order to investigate the genetic difference between the AstV from the chimpanzee in this study and its close relatives from the GenBank, the nearly complete genome was determined by PCR to bridge sequence gaps using PCR primers based on sequences from the library and three closest reference strains from the GenBank. The Sanger method was used to sequence the PCR products.

The nearly complete genome of this AstV is 6120 bp and was named SAstV-nj (GenBank no. KX273058). Genome analysis indicated that, similar to its close relatives in the GenBank, SAstV-nj contained three major ORFs: ORF1a and ORF1b at the 5′ end encoding the non-structural proteins, and ORF2 at the 3′ end encoding the capsid protein. Based on the complete genome, SAstV-nj shared >98% nucleotide sequence identity with three AstV MLB2 strains with have complete genomes available in the GenBank (including JF742759, AB829252 and KT224358). Sequence analysis indicated that, over the ORF1a region (2364 bp), SAstV-nj has 36, 48 and 49 bp differences from those of JF742759, AB829252, and KT224358, respectively. Over the ORF1b region (1536 bp), SAstV-nj has 20, 14 and 22 bp differences from those of JF742759, AB829252, and KT224358, respectively. The ORF2 region (2238 bp) has 25, 41 and 37 bp differences between the SAstV-nj and JF742759, AB829252 and KT224358, respectively.

Phylogenetic analysis was performed based on the sequences of the RNA-dependent RNA polymerase (RdRp) gene and putative capsid protein gene from SAstV-nj, its closest relatives in the GenBank, and other 17 representative AstVs. Results confirmed that SAstV-nj clustered closely with three previous AstV MLB2 strains (Fig. 1b, c), suggesting SAstV-nj belongs to an AstV MLB2 strain.

AstV MLB2 was first detected in human stool samples from India and the United States in 2009 [11]. This virus was subsequently found in febrile children from USA [12], children with diarrhea from Japan and Turkey [13, 14], and patients with meningitis [11]. However, no previous studies mentioned discovering AstV MLB2 from animals. In the present study, an AstV MLB2 strain in a chimpanzee with diarrhea was detected. The viral metagenomic data indicated this chimpanzee was only positive for the AstV MLB2, suggesting that AstV might have potential of cross-species infection between human and NHP and cause diarrhea.

The genome of SAstV-nj was deposited in GenBank under the accession numbers KX273058. The raw sequence reads from the metagenomic library were deposited in the Short Read Archive of GenBank database under accession number SRX2163031.

## Conclusions

Here, we characterized the nearly complete genome of AstV MLB2 strain, named SAstV-nj, from a chimpanzee with diarrhea in China. Sequence and phylogenetic analysis indicated that this AstV MLB2 strain closely clustered with previous human AstV MLB2 strains, sharing >98% sequence identity with them. PCR screening revealed a single positive sample from the 69 NHP fecal samples, suggesting this case of AstV MLB2 infection in the chimpanzee was sporadic. Further studies needs to be performed to confirm whether AstV MLB2 can cause cross-species infection and is associated with diarrhea in non-human mammals.

## Abbreviations

HAstVs: human astroviruses
NHPs: non-human primates
AstVs: astroviruses
NVNR: non-virus-non-redundant
RdRp: RNA-dependent RNA polymerase
